# Environmental Contamination in Relation to cDHP in Candida auris Patient Rooms as Measured by ATPase

**DOI:** 10.1017/ash.2024.240

**Published:** 2024-09-16

**Authors:** Julia Moody, Ken Sands, Bonnie Greene, Rachel Long, Nychie Dotson

**Affiliations:** Hospital Corporation of America Healthcare

## Abstract

**Background:** Candida auris (CA) is an urgent threat per Centers for Disease Control and Prevention with rapidly increasing cases across the US. Patient rooms recontaminate with CA within hours after daily cleaning due to skin shedding, persistence on environmental surfaces and resistance to commonly used disinfectants. Continuous dry hydrogen peroxide (cDHP) is a novel environmental technology augmenting daily room disinfection. cDHP reduces CA organism counts based on environmental cultures. Adenosine triphosphatase (ATPase) testing offers rapid results to monitor surface cleanliness. ATPase Testing Protocol: Upon identification of CA, cDHP was activated in the patient’s room. ATPase surface testing was performed in rooms of CA infected inpatients and nearby control rooms of inpatients without CA and thus no cDHP. Group A surfaces near the patient were nurse call handheld devices and/or bed rail. Group B surfaces were horizontal counter and/or computer keyboard, located >3 feet away from the patient. ATPase testing was to occur within one hour of daily room disinfection for CA patient Day0 (day of cDHP activation), Day1, Day7 and Day14 and controls. Daily room disinfection using quaternary disinfectants was replaced with EPA Class P chemicals upon CA identification. Nursing spot disinfects with Class P ready to use disinfectant wipes in all rooms. **Results:** Testing occurred among 13 CA and 22 control patients in 5 hospitals. In Table 1, pass rates are displayed by cumulative (Day0+1+7+14) test days for surfaces and patient room groups. Analysis applied Pearson’s Chi-squared test with Yates’ continuity correction. **Conclusions:** Surfaces further from the patient in rooms of CA patients exposed to cDHP had higher ATPase pass rates than controls. Surfaces close to the patient have a high ATPase failure rate, regardless of CA or cDHP. Strategies are needed to ensure disinfection occurs on high touch surfaces near patients. cDHP may have value in supplementing room disinfection. Contributing failure factors include surfaces missed for disinfection, delays in timely testing and known limitations with ATPase methods.

